# The new perspective on understanding the mechanisms of cardiovascular diseases development

**DOI:** 10.1038/s41598-025-11345-8

**Published:** 2025-07-22

**Authors:** Ulyana Khovantseva, Diana Kiseleva, Vadim Cherednichenko, Deyyara Chakal, Denis Breshenkov, Yuliya Markina, Rustam Ziganshin, Eduard Charchyan, Alexander Markin

**Affiliations:** 1 Petrovsky National Research Center of Surgery, 119435 Moscow, Russian Federation; 2 Petrovsky Medical University, 119435 Moscow, Russian Federation; 3 Lomonosov Moscow State University, 119991 Moscow, Russian Federation; 4 Shemyakin-Ovchinnikov Institute of Bioorganic Chemistry Russian Academy of Sciences, 117997 Moscow, Russian Federation

**Keywords:** Cardiovascular diseases, Smooth muscle cells, Macrophages, Phagocytosis, Liquid chromatography–mass spectrometry, Cell biology, Cardiology

## Abstract

Cardiovascular diseases (CVD) are one of the leading causes of death worldwide. From a modern point of view, endothelial dysfunction is considered as a key factor leading to the development of CVD. However, scientists have suggested that the main causes of the development of CVD might be functional disorders and phenotypic modulation of smooth muscle cells (SMCs) that make up the vascular wall. In this regard, the aim of our study was to evaluate the functional features of SMCs isolated from the tunica intima and from the tunica media of the thoracic part of the human aorta in patients with CVD. In our research we showed that phenotypic switching can occur in SMCs isolated from patients with aneurysms (n = 6), resulting in remodeling of the extracellular matrix and impaired interaction between cellular receptors. In addition, it is probable that the activation of complement-mediated phagocytosis as a result of LDL internalization by SMCs might be one of the key mechanisms in the process of aneurysm development.

## Introduction

It is well known that cardiovascular diseases (CVD) are one of the leading causes of death worldwide. More than 17.9 million people die from CVD every year^1^, however, the cellular and molecular mechanisms of CVD development remain not fully understood. From the generally accepted point of view endothelial dysfunction is considered as a key factor leading to the development of CVD^2^. However, it has recently been suggested among scientists that the cause of the development of CVD might be the phenotypic switching of smooth muscle cells (SMCs) that constitute the most abundant cell type in the vascular wall^3^.

Vascular SMCs are the predominant cell type in the middle aortic layer—the tunica media. The main function of SMCs is to regulate vascular tone due to their ability to contract. Impaired SMCs contractility leads to the development of various CVD, for example, aneurysm. SMCs apoptosis might be an early sign of CVD development. This, in turn, leads to a SMCs density decrease and a decrease in the secretion of extracellular matrix (ECM), which may result in the dilation of the vessel wall and the progression of CVD^4^. The previously described phenomenon of switching SMCs phenotypes between contractile (“normal” phenotype) and synthetic (“pathological” phenotype) in response to pathological stimulation might be the cause of SMCs dysfunction development^5^. Typical SMCs are characterized by a powerful contractile apparatus (smooth muscle actin αSMA and myosin heavy chain SM-MHC)^6^, whereas in the synthetic SMCs, the expression of these proteins is lower.

In addition, with the development of CVD, SMCs from tunica media can migrate to the subendothelial space of tunica intima, where they proliferate and produce ECM^7^. This phenomenon suggests that SMCs can modulate its phenotype to a pathological one, which can lead to the development of cardiovascular diseases, such as atherosclerosis^8^. Atherosclerosis is characterized by narrowing of the vascular lumen, due to the formation of atherosclerotic plaques. Atherosclerotic plaques consist of a lipid-necrotic nucleus and a cap. The plaque cap has a heterogeneous structure, which consists of SMCs, collagen fibers, immunocompetent cells (macrophages, mast cells), and endothelial cells^9^. In addition, a cenumber of studies have shown that macrophages, which are part of atherosclerotic plaques in patients with atherosclerosis, can actively internalize low-density lipoproteins (LDL). As a result, macrophages begin to actively secrete pro-inflammatory factors (IL-6, IL-8, TNF-α)^10^, that worsen the development of CVD.

Thus, it is assumed that dysregulation of phenotypic switching is a key process in the formation of CVD, but the mechanisms of this process are still not fully understood. Recent transcriptomic studies have shown that SMCs of the aortic wall are a much more dynamic cell population than previously thought^11^. Single-cell transcriptomic analysis has shown that the pathological phenotype of SMCs is represented by a set of heterogeneous cell populations. Among them are contractile, macrophage-like, mesenchymal-like, fibroblast-like, osteogenic-like, and adipocyte-like SMCs^12,13^.

The aim of this study was to evaluate the functional features of smooth muscle cells isolated from tunica intima and tunica media of the thoracic part of the human aorta for patients with CVD. At the moment, there are no necessary and suitable laboratory tools for controlled phenotypic switching within the vascular SMCs population. Therefore, in our work, a linear monocyte cell culture corresponding to the macrophage-like SMCs phenotype detected by single-cell transcriptome analysis was used as an internal control to compare the characteristics of primary SMCs in CVD patients.

## Results

### The phagocytic activity and the ability to internalize LDL

**Fig. 1:**
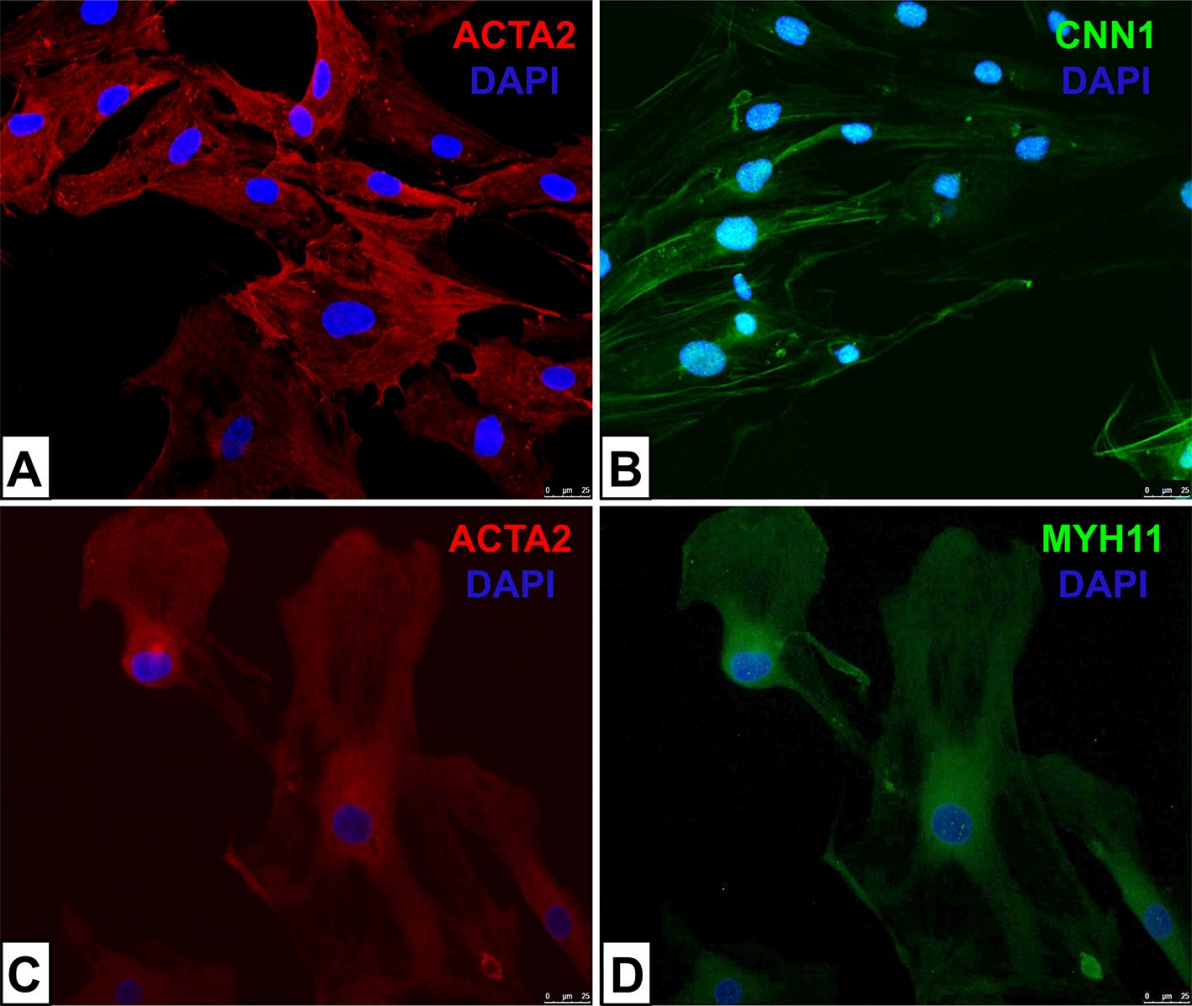
The immunofluorescence of smooth muscle alpha-actin-2 (ACTA2), calponin 1 (CNN1) and the smooth muscle myosin heavy chain in primary smooth muscle cells isolated from the human aorta. (**A**) SMCs from the tunica media were fixed and incubated with ACTA2 antibody (red). (**B**) SMCs from the tunica media CNN1 were fixed and incubated with CNN1 antibody (green). (**C**) SMCs from the tunica intima were fixed and incubated with ACTA2 antibody (red). (**D**) SMCs from the tunica intima were fixed and incubated with MYH11 antibody (green). Nuclei were stained with DAPI (blue) (**A**–**D**). The scale bar is 25 microns (**A**–**D**).

In our study we showed that the studied cell lines have markers of SMCs, namely: aortic smooth muscle alpha-actin-2 (ACTA2), calponin 1 (CNN1) and the smooth muscle myosin heavy chain (MYH11) (Fig. 1).

We used these proteins because they are markers of SMCs: ACTA2 is involved in the contractile apparatus of SMCs^14^, CNN1 is a basic smooth muscle protein^15^ and MYH11 is an essential contractile protein within SMCs^16^.

Studied cell lines have different sizes: macrophages—about 21 µm^17^, SMCs about 100 – 200 µm^18^. Consequently, for statistical analysis, the number of phagocytized beads and internalized LDL in cells was calculated per µm^2^.

The studied statistical analysis of cell lines phagocytic activity showed that the average number of absorbed latex beads in cells per µm^2^ was: for macrophages 0.055 ± 0.022 items.; for SMCs from the tunica intima 0.019 ± 0.009 items.; for SMCs from the tunica media 0.025 ± 0.018 items.

**Fig. 2:**
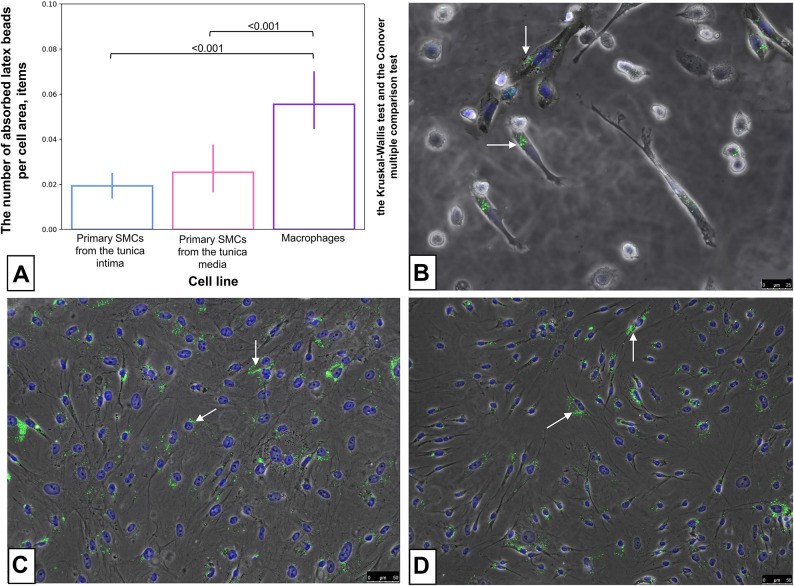
The level of statistical significance of intergroup differences in the studied groups after incubation of the cells with latex beads (**A**) and studied cell lines after incubation with latex beads (0.5 µm) (**B**,**C**,**D**). Cells were treated with latex beads (green, indicated by arrows) for 2 h to induce phagocytosis. Cells were fixed and stained with DAPI (blue). (**B**) macrophages. (**C**) SMCs from the tunica intima. (**D**) SMCs from the tunica media. B- the scale bar is 25 microns. (**C**,**D**) the scale bar is 50 microns.

Macrophages showed a statistically higher level of phagocytosis compared to other cell lines (p < 0.001) (Fig. 2).

The obtained data might indicate that SMCs isolated from the tunica intima and SMCs isolated from tunica media of aneurysm patients increase the rate of endocytosis in vitro, so they can actively absorb latex beads.

**Fig. 3:**
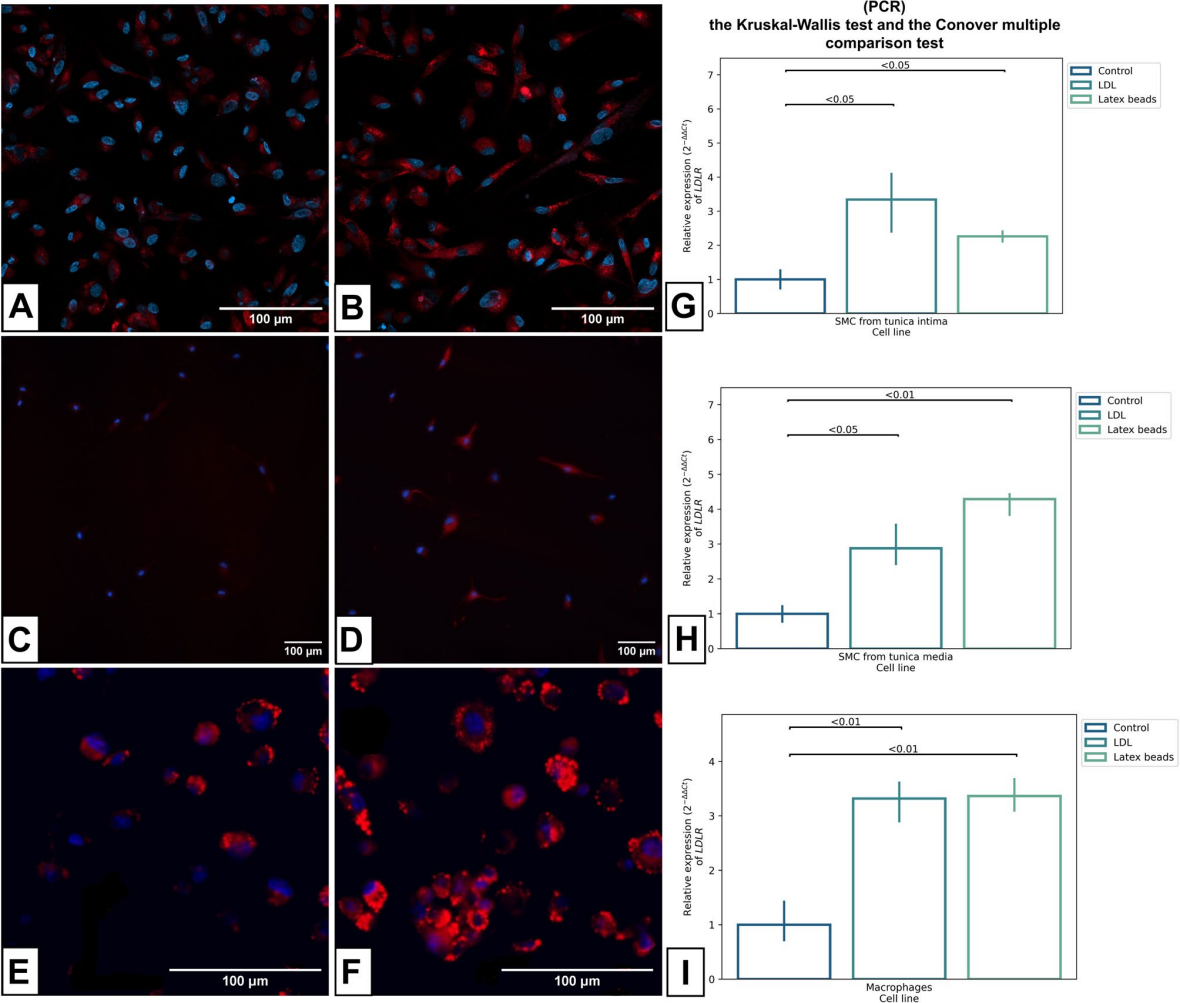
BDP 630/650 staining of lipid droplets (**A**–**F**) and the relative expression of the *LDLR* gene (**G**–**I**) in studied cell lines. In experimental groups (**B**,**D**,**F**) cells were treated with LDL for 24 h to induce intracellular lipid accumulation. In control groups (**A**,**C**,**E**) studied cells were cultured in a medium without LDL and fetal bovine serum. Cells in control and experimental groups were fixed and stained with BDP 630/650 (red) and DAPI (blue). (**A**,**B**) SMCs from the tunica intima. (**C**,**D**) SMCs from the tunica media. (**E**,**F**) macrophages. The scale bar is 100 microns (**A**–**F**). (**G**) the relative expression of the *LDLR* in SMCs from tunica intima. (**H**) the relative expression of the *LDLR* in SMCs from tunica media. (**I**) the relative expression of the *LDLR* in macrophages. The relative expression of the *LDLR* in the studied cell lines was calculated using the 2^−ΔΔ*Ct*^ method (**G**–**I**).

The analysis of the ability of cell lines to internalize LDL showed that in cell lines without LDL, the average fluorescence intensity of the BDP dye per µm^2^ in the cell was significantly lower than the one after incubation with LDL (Fig. 3A–F, Table 1).

**Table 1:** The median values of the relative index of the normalized fluorescence intensity of the BDP 630/650 dye in the studied cell lines per µm^2^ in cell, a.u., Me (25th; 75th percentile).

Cell line	Macrophages	SMCs from the tunica intima	SMCs from the tunica media
Without LDL, × 10^–4^	8 (6;8)	0.6 (0.4;0.8)	0.2 (0.1;0.4)
With LDL, × 10^–4^	20 (20;20)	1 (0.8;1.3)	1 (0.9; 2)
Significance, p	< 0.001	< 0.01	< 0.001

Table 1 shows that the accumulation of cholesterol in SMCs isolated from the tunica intima and in SMCs isolated from the tunica media is lower than that in macrophages. This is due to the fact that SMCs are not professional phagocytes, but they can acquire a macrophage-like phenotype, as a result of which they accumulate lipid droplets but not as actively as macrophages.

In addition, to confirm that phagocytosis in the studied cell lines occurs via the LDLR (LDL receptor), we performed RT-qPCR to assess the level of LDLR expression. As a result of the PCR analysis, it was shown that the expression of the LDLR significantly increases in the studied cell lines in the groups of cells after incubation with LDL and latex beads compared with the control groups (Fig. 3G-I).

After that, we analyzed the culture medium of the studied cells using chromatography-mass spectrometry.

### The proteomic analysis of the studied cells secretome using LC–MS

**Fig. 4:**
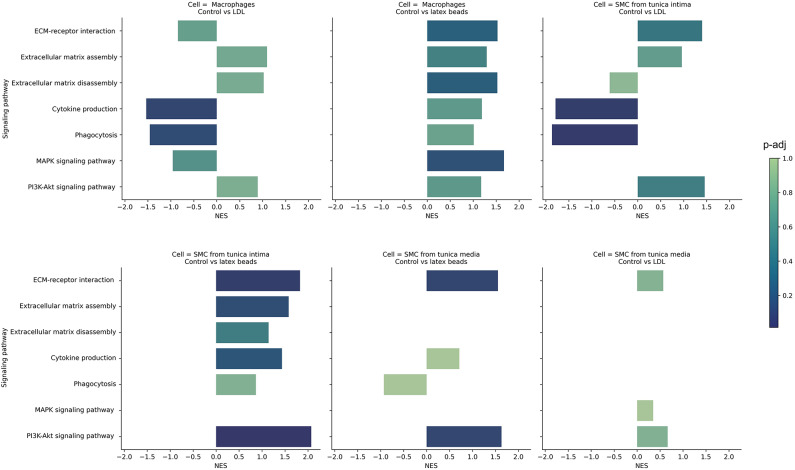
GSEA for protein lists ranked according to the statistics obtained for macrophages control vs LDL, macrophages control vs latex beads, SMCs from tunica intima control vs LDL, SMCs from tunica intima control vs latex beads, SMCs from tunica media control vs latex beads, SMCs from tunica media control vs LDL. Normalized enrichment score (NES) is on the x-axis, the scale bar reflects the adjusted p-value (p-adj). The results are shown for the KEGG pathway database.

During the proteomic analysis of the secretome, it was shown that the process of phagocytosis of latex beads and LDL internalization occurs through various signaling pathways in the studied cell lines, where various proteins are involved (Fig. 4).

Thus, in our research, we showed that during phagocytosis of latex beads in SMCs isolated from the tunica intima, the ECM remodeling pathway is inhibited, namely, the ECM assembly compared with the control group of cells. Statistically significant proteins that probably trigger the signaling pathway of ECM assembly in the control group of SMCs isolated from the tunica intima are: COL6A1 (Collagen Type VI Alpha 1Chain), COL6A2 (Collagen Type VI Alpha 2 Chain) and FBLN5 (Fibulin 5) (p < 0.05). All of these proteins are the main components of microfibrils^19^, which are components of the ECM^20^. In the macrophages and SMCs isolated from the tunica media after the absorption of latex beads, the extracellular matrix assembly and disassembly pathways are inactive. We did not find statistically significant differences in SMCs isolated from the tunica media in the studied signaling pathways.

We also showed that during the absorption of latex beads in SMCs isolated from the tunica intima and in SMCs isolated from the tunica media, there is a disruption in the signaling pathways of interaction between ECM and receptors on the cell surface (ECM receptor interaction), as well as the PI3K/Act signaling pathway, which are interconnected. Thus, in SMCs isolated from the tunica intima, a statistically significant decrease in proteins was shown: COL1A1, COL1A2, COL6A1, COL6A2, THBS2 (Thrombospondin 2) and FN1 (Fibronectin 1) in the group of cells after incubation with latex beads, compared with the control group of cells (p < 0.05). In SMCs from the tunica media, a significant decrease in the secretion of proteins THBS2, COL6A1, COL6A2 and FN1 was found in the group of cells after incubation with latex beads, compared with the control group of cells (p < 0.05). It is worth noting that collagens are extracellular matrix proteins^21^, while THBS2 and FN1 proteins mediate intercellular interactions^22,23^, that is why a decrease in their number might be one of the main reasons leading to a violation of the interaction between ECM and receptors on the cell surface.

Moreover, in macrophages after incubation with balls, on the contrary, the PI3K/Act signaling pathway is active but the MAPK signaling pathway is inactivated, in which statistically significant differences were found in the proteins CSF1 (Colony stimulating factor 1), CSF1R (Colony stimulating factor 1 receptor) and TGFB1 (Transforming growth factor beta1), compared with the control group of cells (p < 0.05). This might be due to the fact that all these proteins are associated with receptor tyrosine kinases, which are the main participants in MAPK signaling pathways and they mediate intercellular communication.

**Fig. 5:**
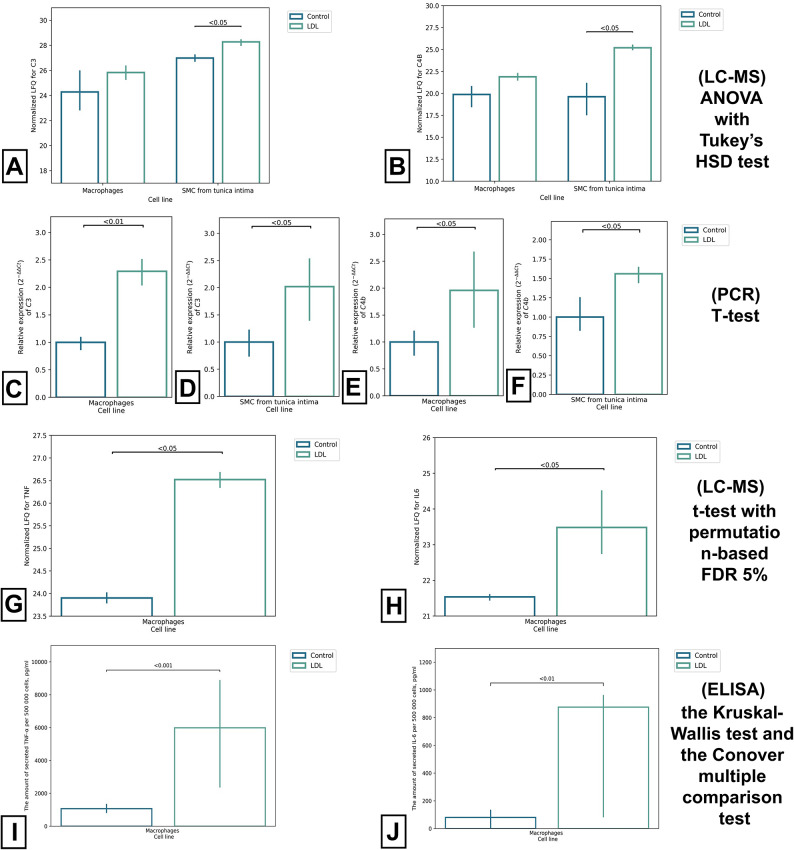
The proteins of the complement system in the study cell lines and pro-inflammatory cytokines secreted by macrophages. (**A**) secretion of C3 protein in studied cell lines, (**B**) secretion C4B protein in studied cell lines. (**A**,**B**) log2 normalized LFQ Intensity is shown on the y-axis. (**C**) expression of C3 in macrophages, (**D**) expression of C4B in SMCs from the tunica intima, (**E**) expression of C4B in macrophages, (**F**) expression of C4B in SMCs from the tunica intima. (**C**,**D**,**E**,**F**) PCR analysis. (**G**) secretion of TNF in macrophages (LC–MS), (**H**) secretion of IL-6 in macrophages (LC–MS). (**G**,**H**) log2 normalized LFQ Intensity is shown on the y-axis. (**I**) secretion of TNF in macrophages (ELISA). (**J**) secretion of IL-6 in macrophages (ELISA).

During the proteomic analysis, we also showed that the internalization of LDL by SMCs isolated from the tunica intima and macrophages activates the phagocytosis signaling pathway. It was shown that after incubation with LDL in a culture medium from SMCs isolated from the tunica intima, the content of complement system proteins increases: C3 and C4B, compared with the control group (p < 0.05) (Fig. 5A,B). It is important to note that as a result of PCR analysis, we showed that in SMCs from tunica intima after incubation with LDL, there is a significant increase in the expression of genes: *C3* and *C4B* compared with the control group (p < 0.05) (Fig. 5D,F), which confirms the results obtained during LC–MS. PCR analysis also revealed that the expression of *C3* and *C4B* in the control group of macrophages was significantly lower than in the group after incubation with LDL (p < 0.05) (Fig. 5C,E).

However, we have not found statistically significant differences between proteins potentially activating the phagocytosis signaling pathway in macrophages after incubation with LDL compared with the control group.

In addition, the production of cytokines in SMCs isolated from the tunica intima decreases in the group after incubation with latex beads, and this might be due to the FN1 protein since its content in the control group of cells is higher than in the group of cells after incubation with latex beads (p < 0.05). However, it is worth noting that incubation with LDL, on the contrary, activates the secretion of cytokines in SMCs isolated from the tunica intima and in macrophages. Thus, in SMCs isolated from the tunica intima, the secretion of the following proteins increased: C3, SAA1 (Serum amyloid A1) and ORM (Orosomucoid) compared with the control group (p < 0.05). While the secretion of proinflammatory cytokines TNF and IL-6 increased in the THP-1 professional phagocyte cell line in the group after incubation with LDL, compared with the control group (p < 0.05) (Fig. 5G,H).

However, due to the low secretion of IL-6, IL-8, TNF-α, and IL-10 in primary SMCs, it was not possible to measure their concentrations using LC–MS, as they did not pass the sensitivity threshold of the device. In this regard, we used ELISA to measure the amount of secreted cytokines in SMCs (Fig. 5I,J). Macrophages were used as a reference group to confirm the results obtained by LC–MS.

### Analysis of cytokine secretion in the studied cells using ELISA

Analysis of the culture medium revealed that the secretion of pro-inflammatory (IL-6, IL-8, TNF-α) and anti-inflammatory (IL-10) cytokines differs significantly between different cell groups. During the study, we analyzed 3 cell lines, which included 3 groups for each cell line. We conducted three repeated experiments for each cytokine.

The results of measurements of IL-6, IL-8, TNF-α, and IL-10 secretion in the studied cell lines in the groups after incubation with LDL and latex beads, as well as in the control, are presented in Table 2.

**Table 2:** The median value of the absolute index of secreted cytokines (IL-8, IL-6, IL-10, TNF-α) in the studied cells, pg/ml, Me (25th; 75th percentile).

Secreted cytokine	Cell line	Group			Significance, p
		Control (1)	LDL (2)	Latex beads (3)	
IL- 8	Macrophages	97,900 (73,280; 115,559)	96,882 (72,372; 113,689)	13,932 (11,719; 1595)	p_(1–3)_ < 0.05 p_(2–3)_ < 0.05
	SMCs from the tunica intima	169 (155; 180)	423 (383; 487)	514 (415; 585)	p_(1–2)_ < 0.01 p_(1–3)_ < 0.01
	SMCs from the tunica media	526 (410; 647)	450 (318; 553)	847 (549; 1033)	p_(2–3)_ < 0.05
IL-6	Macrophages	80 (16; 118)	876 (285; 904)	5 (4; 12)	p_(1–2)_ < 0.01 p_(1–3)_ < 0.01
	SMCs from the tunica intima	1439 (1027; 1973)	1181 (1107; 1826)	135 (126; 410)	p_(1–2)_ < 0.01 p_(2–3)_ < 0.01
	SMCs from the tunica media	1862 (1488; 1995)	1247 (1078; 1874)	2026 (1151; 2279)	p > 0.05
IL-10	Macrophages	170 (128; 1003)	183 (149; 1084)	208 (176; 1105)	p > 0.05
	SMCs from the tunica intima	130 (108; 1367)	132 (129; 1456)	168 (165; 1279)	p > 0.05
	SMCs from the tunica media	117 (98; 123)	126 (104; 128)	126 (123; 146)	p > 0.05
TNF-α	Macrophages	1066 (910; 1287)	5987 (3682; 6987)	970 (718; 1194)	p_(1–2)_ < 0.001 p_(2–3)_ < 0.001
	SMCs from the tunica intima	141 (135; 145)	141 (126; 146)	152 (134; 161)	p > 0.05
	SMCs from the tunica media	131 (128; 147)	142 (116; 144)	145 (132; 150)	p > 0.05

According to the obtained data, secretion of IL-8 in SMCs isolated from the tunica intima after incubation with LDL was significantly lower than in the group of cells without LDL (p < 0.001). The secretion of IL-8 in SMCs isolated from the tunica media was significantly higher in the group after incubation with latex beads compared with the group of cells after incubation with LDL (p < 0.05). The obtained data demonstrate that the secretion of proinflammatory cytokines increases in SMC isolated from the tunica intima and in SMCs from the tunica media of patients with CVD after proinflammatory activation.

Our study also showed higher IL-6 secretion in the group of cells after incubation with LDL compared with the control group and the group after incubation with latex beads. Thus, IL-6 secretion in macrophages after incubation with LDL was significantly higher than in the control group (p < 0.001) and in the group after incubation with latex beads (p < 0.001). These data confirm the results obtained during LC–MS analysis. There were no statistically significant differences in IL-6 secretion in SMCs isolated from the tunica intima without the addition of agents and after incubation with LDL.

It is worth noting that TNF-α secretion in macrophages after incubation with LDL was significantly higher than in the control group of cells (p < 0.01) and the group of cells after incubation with latex beads (p < 0.01), which confirms the results obtained during LC–MS analysis. No statistically significant differences in TNF-α secretion were found between the groups within the SMCs isolated from the tunica intima and in the SMCs from the tunica media. No statistically significant differences in TNF-α secretion were found between the groups within the SMCs isolated from the tunica intima and in SMCs from the tunica media.

We found no statistically significant differences in the secretion of anti-inflammatory IL-10 in the studied cell lines.

## Discussion

The role of SMCs during the development and progression of aortic aneurysms is not well defined. In this study, we have shown that switching of SMCs phenotype can lead to the development of a thoracic aortic aneurysm. We have demonstrated that this phenotypic switch can occur in response to the action of inflammatory factors, such as LDL and latex beads. Thus, increased phagocytic activity and an increase in LDL levels leads to various disorders in the signaling pathways presumably involved in the development of aneurysms and, probably, the activation of complement-mediated phagocytosis. All this contributes to an increased secretion of pro-inflammatory cytokines by SMCs and various functional disorders, and, as a result, the development of a thoracic aorta aneurysm. Our findings support that the phenotypic modulation of SMCs has an importance role in the development of CVD.

It is known that the LDL absorption process occurs with the help of the LDLR, which recognizes apolipoprotein B, which is part of LDL^24^. Despite the fact that the role of LDLR in regard to lipids is well known, its role in the development of CVD has not been sufficiently studied^25^. In our research, we showed how the internalization of LDL by cells that form the structure of the aortic wall affects the development of aneurysms. The work demonstrated that the cholesterol accumulation process increases the secretion of IL-6 and IL-8 in the studied cell lines. This is due to the fact that LDL particle endocytosis is mediated by LDLR^26^. Our data demonstrated that in the studied cells, after incubation with LDL, there is an increase in LDLR expression. This confirms the assumption that with elevated cholesterol levels, LDLR binds LDL particles on the cell surface and releases them into endosomes, during which the NF-kB pathway is activated^27^. This activation triggers the synthesis of pro-inflammatory cytokines, chemokines, and adhesion molecules^28^. This might be due to the fact that with the development of CVD, the SMCs of the aorta acquire a phenotype associated with the secretion of pro-inflammatory cytokines. This confirms that the phenotypic switching of SMCs might be a key factor in the development of inflammation inside the vessel membrane^6^.

Moreover, in our study, we showed that during the internalization of LDL, SMCs isolated from the tunica intima begin to actively secrete proteins of the complement system: C3, C4B, while this was not detected in macrophages. At the same time, PCR analysis showed that expression of *C3* and *C4B* genes increases in both SMCs isolated from the tunica intima and macrophages after incubation with LDL, compared with the control groups. It is known that anaphylatoxins released during the complement system activation are involved in the regulation of inflammatory reactions and therefore in the development of various diseases^29^. However, the role of the complement system in the development of CVD is still unknown. Recent studies have shown that patients with thoracic aortic dissection have elevated levels of C3 and C4 in blood plasma^30–32^. This might be due to the fact that in SMCs in patients with CVD, the floating of C3 protein increases during LDL internalization, as a result of which complement-mediated phagocytosis is activated. During complement-mediated phagocytosis, the C3 protein causes a SMCs contraction, increases vascular permeability, and leads to the release of histamine from mast cells^33^. As a result, SMCs begin to actively secrete pro-inflammatory cytokines, which leads to the development of inflammation^34^. At the moment, there is no accurate data on how exactly the complement system affects the development of an aneurysm. However, scientists have shown that with the development of an abdominal aortic aneurysm, the lectin and classical pathways of the complement system are activated, and inhibition of their activation prevents the development of an aneurysm^35^. In addition, in a study on rats, it was shown that an increase in the expression of the C3 gene induces a switch to the synthetic phenotype of SMCs^36^. Our data also demonstrate that in smooth muscle cells of people with aneurysms, there is an increase in the expression of the *C3* gene, which leads to the phenotypic modulation of SMCs to a synthetic phenotype and the development of inflammation. We were also interested in another study, where it was shown that in a THP-1 cell line incubated with LDL after 24 h, a certain expression of the *C3* gene was observed and the secretion of the C3 protein decreased^37^. Our results also demonstrate that after 24 h of incubation with LDL in macrophages, there is a decrease in the secretion of proteins of the complement system (C3, C4B) and an increase in gene expression: *C3* and *C4B*. This might be due to disturbances in post-translational and post-transcriptional regulation^38,39^, which can eventually lead to impaired antigen presentation by macrophages and, consequently, the development of inflammation. Moreover, in our work, we have shown that SMCs isolated from the tunica intima actively secrete proteins C3 and C4B, therefore, it can be assumed that the classical and alternative pathways of the complement system are activated with thoracic aortic aneurysm. The alternative pathway differs from the classical one in that the activation of the complement system does not require the formation of immune complexes, and its activators can be bacteria or tumor cells^40^. In a recent study of aortic aneurysms in mice with Marfan syndrome, scientists have shown that inhibition of C3a/C3aR complement attenuates aneurysm formation^41^. Thus, the activation of complement-mediated phagocytosis as a result of LDL internalization probably might be one of the key mechanisms in the development of CVD, and the activation of the classical and alternative pathways of the complement system plays a key role in the formation of thoracic aortic aneurysms. Based on this, the inhibition of the complement system activation might be a potential target for the treatment of thoracic aortic aneurysm.

It is known that macrophages play an important role in the inflammatory response by phagocytizing tissue debris, LDL and necrotic cells^42^. However, the present study demonstrated the high ability of the studied aortic SMCs in patients with CVD to phagocytosis. The main participants in the phagocytosis process are phagosomes and phagolysosomes, which include about 600 different proteins^43^. However, to date, the molecular mechanisms involved in the process of phagocytosis and cell surface remodeling have not been fully studied. The specific pathway phagocytosis takes place and the membrane proteins involved in it depends on the type of cells that participate in the phagocytosis process and the size of the absorbed particles. To date, there are several proposed pathways for the phagocytosis of latex beads and the activation of secretion of proinflammatory molecules. There is a study where scientists say that the mechanism of endocytosis will depend on the size of the latex beads that the cell absorbs^44^. So, if the latex beads are less than 200 microns, then absorption will follow the clathrin-mediated pathway. If the size of the latex beads is more than 200 microns, then absorption occurs due to a mechanism based on internalization mediated by caveolae^45^. Such as caveolae contain sphingolipids such as cholesterol, LDL internalization may occur when latex particles are absorbed^46^, during which LDLR activation will occur and, consequently, cytokine secretion, along the pathway described above. The size of the latex beads we used in our work was 500 microns. The study showed that SMCs isolated from the tunica intima after incubation with latex beads actively secrete pro-inflammatory cytokines. This confirms that the absorption of particles larger than 200 microns by the cell is due to a mechanism based on the internalization mediated by caveolae. However, the secretion of pro-inflammatory cytokines in the THP-1 cell line in the group after incubation with latex beads had no statistical differences compared with the control group. This might be due to the fact that lipid rafts are located in the membrane of macrophages M1 and M2, which contain scavenger receptors scavenger receptor (SR), the activation of which leads to the development of a pro-inflammatory cell response. When latex beads interact with SR, the actin cytoskeleton of the cell is rearranged, which leads to phagocytosis, during which the inflammasome is activated and the cells begin to actively secrete pro-inflammatory cytokines^47^. In our study, we activated the THP-1 monocyte cell line using PMA to differentiate them into M0 macrophages. Since M0 macrophages do not have SR in their structure^48^, the activation of which leads to a proinflammatory cell response, it can be assumed that phagocytosis proceeds by a mechanism during which pro-inflammatory cytokines are not secreted. In addition, the balance between the secretion of pro- and anti-inflammatory cytokines plays an important role in the development of CVD. The development of inflammatory processes, extracellular matrix degradation, and vascular cell apoptosis in CVD depends on the amount of cytokines secreted^49^. In our study, we measured IL-10 secretion in all the studied cell lines. We have shown that in cells after incubation with LDL or microspheres, IL-10 secretion increased, but only slightly. This may indicate that IL-10 has an anti-inflammatory effect in the development of CVD^50^.

Also, the study showed that different proteins are involved in the process of phagocytosis of latex beads in the cell line of professional phagocytes THP-1 and in SMCs of the human aorta, and therefore the process of absorption of microspheres in the studied cell groups triggers different signaling pathways. Thus, we have shown that there is a decrease in the secretion of proteins: COL1A1, COL1A2, COL6A1, COL6A2, THBS2, and FN1 during phagocytosis of latex beads in SMCs isolated from the tunica intima. In SMCs from the tunica media, as well as in SMCs isolated from the tunica intima, the latex beads absorption reduces the number of proteins: COL6A1, COL6A2, THBS2, and FN1. All of these proteins are associated with the signaling pathway of interaction between ECM and receptors on the cell surface (ECM receptor interaction). It is known that SMCs are normally located in the tunica media of the human aorta, however, with the development of inflammation, they can migrate to the tunica intima, which is associated with the remodeling of the ECM^51^. Based on the data we have obtained, we can say that the key proteins involved in the process of extracellular matrix remodeling are COL1A1, COL1A2, COL6A1, COL6A2, and probably a decrease in their secretion during phagocytosis of latex beads is a key process leading to disruptions in the interaction between ECM and receptors on the cell surface. In addition, these changes can lead to the inactivation of the PI3K/AKT signaling pathway in SMCs isolated from the tunica intima and in SMCs isolated from the tunica media, which normally participates in maintaining the normal functioning of the cell cycle^52^. Thus, we can assume that during the development of CVD, the mechanisms of interaction between ECM and receptors on the cell surface are disrupted, which leads to the PI3K/ACT pathway inactivation, and consequently to the aortic SMCs cell cycle disruption.

We did not find the effect of phagocytosis of latex beads on changes in ECM in macrophages. It is known that CSF 1 and CSF1R proteins, which are receptors for colony-stimulating factor 1^53^, as well as the TGFB protein, play an important role in the proliferation and differentiation of mononuclear phagocytes, including THP-1^54^. Scientists suggest that inhibition of the TGF-β signaling pathway contributes to the development of CVD^55^. Our results confirm this assumption. So, in our study, we showed that during phagocytosis of latex beads, there is a decrease in the secretion of proteins: CSF1, CSF1R and TGFB1. Normally, the action of stress factors leads to the activation of tyrosine kinase receptors, where the CSF1 receptor can act as a signaling molecule, as well as to the TGFB receptor activation, where the transforming growth factor TGFB is the signaling molecule. Activation of these receptors leads to the launch of the MAPK signaling pathway and the maintenance of normal cell function^56^. However, phagocytosis of latex particles by professional phagocytes THP-1 led to the inhibition of the MAPK signaling pathway and, as a result, to the disruption of the normal functioning of macrophages.

Thus, the phagocytosis of latex beads disrupts the cell cycle in the SMCs of the aorta and macrophages, as a result of which cells can change their normal structure and function, which might be one of the key molecular mechanisms in the CVD development. In addition, it can be assumed that PI3K/AKT and MAPK signaling pathways can be used as drug targets for the treatment of CVD.

Our study has limitations. In our work, we studied SMCs from the thoracic aorta. This is due to the fact that patients with abdominal aortic aneurysm frequently have atherosclerosis^57^. Because of this, the intima of the abdominal aorta becomes thinner and contains many atherosclerotic lesions and calcifications, which makes it difficult to isolate SMCs. However, it would also be interesting to study the abdominal aortic SMCs, since thoracic and abdominal aortic aneurysms have striking similarities on a general anatomical level, and the underlying pathophysiology has quite distinct differences^58^.

It is also worth mentioning that in our work we used the THP-1 cell line as a control. Although this cell line is a generally accepted model, it is important to understand its limitations. Since the THP-1 cell line was obtained from a sample of a patient with acute myeloid leukemia, its ability to fully differentiate and acquire all the properties of mature macrophages of the immune system might be limited, which can affect the results of studying normal physiological processes. In addition, the experimental results can depend on the cultivation conditions, such as the composition of the medium, the concentration of stimuli, and the incubation conditions. This requires careful standardization of protocols and consideration of possible effects of cultivation conditions on the results.

In our study, we initially tested our hypothesis. However, further studies will need to be conducted to confirm the molecular data and verify the specific mechanisms involved in aneurysm progression. For example, Western blotting can be performed to supplement the data obtained on the components of the complement system (C3, C4B) and inflammatory cytokines (IL-6, IL-8, TNF-α and IL-10). Additionally, an experiment on single-cell sequencing can also be used to confirm our results on the phenotypic modulation of SMCs.

Thus, the phenotypic switching of smooth muscle cells from a normal (contractile phenotype) to a pathological phenotype (synthetic or macrophage-like) might be one of the main pathological processes causing cardiovascular diseases, including thoracic aortic aneurysm, but the specific molecular mechanisms and signaling pathways involved can differ depending on the disease. The phenotypic modulation of smooth muscle cells can be the result of remodeling of the extracellular matrix and disruption of the interaction between cellular receptors. As a result, smooth muscle cells begin to actively secrete pro-inflammatory cytokines, which leads to the development of local inflammation in the vessel wall. In addition, it is likely that the activation of complement-mediated phagocytosis as a result of the internalization of low-density lipoproteins by smooth muscle cells is one of the mechanisms involved in the development of cardiovascular diseases. The activation of classical and alternative pathways of the complement system might be one of the important processes in the formation of thoracic aortic aneurysms. Based on this, the inhibition of the complement system activation and the phenotypic switching of smooth muscle cells might be potential targets for the treatment of aneurysms. The obtained data about functional capabilities of the studied cells expand our knowledge about the importance of smooth muscle cells in the development of cardiovascular diseases and are a prerequisite for future research.

## Materials and methods

### Ethical expertise

The study protocol was approved by the Local Ethics Committee of Petrovsky National Research Center of Surgery, Moscow, Russia (No. 8, October, 20, 2022). The study was conducted in accordance with the Declaration of Helsinki of 1975 and its revised version of 2013. All the participants signed written informed consent to participate in the study.

### Studied material

The main object of the study were SMCs isolated from the intima and media of the thoracic aorta in patients with aortic aneurysm. The study included patients with thoracic aortic aneurysm (n = 6) (Table 3) requiring surgical treatment and involving the manipulation of aortic tissues and the explantation of its affected segments.

**Table 3:** Characteristics of the studied material.

Number	Sex	Age	Diagnosis
1	Male	57	Aortic root and ascending aorta aneurysms
2	Male	67	Aortic root and ascending aorta aneurysms
3	Female	61	Aortic root and ascending aorta aneurysms. Severe aortic regurgitation. Shaggy aorta
4	Male	54	Aortic root and ascending aorta aneurysms. Severe aortic regurgitation. Marfan syndrome
5	Male	43	Thoracic aortic aneurysm. Aortic valve regurgitation
6	Male	32	Aortic Root and Ascending Aorta Dilatation. Severe aortic regurgitation. Moderate aortic stenosis

Taking into account the general nosology of patients, in these groups, there was no statistically significant influence of gender and age, and the features of the disease course on the studied parameters.

In our study, we used the cell line of macrophages (PMA (phorbol-12-myristate-13-acetate)-induced THP-1 macrophages (ATCC, Manassas, Virginia, USA)) and primary SMCs isolated from the tunica intima and SMCs isolated from the tunica media of human aorta in patients with CVD. We utilized THP-1 professional phagocyte cell line as the reference group^59^. THP-1 is a suspension human leukemia monocytic cell line, which has been extensively used to study macrophage functions, mechanisms and signaling pathways in the cardiovascular system^60^. THP-1 cells exhibit a large, round, single-cell morphology. They can be differentiated into macrophage-like cells with treatments like phorbol esters. Moreover, THP-1 cells are phagocytic, meaning they can engulf and internalize particles, which is a crucial function of macrophages^61^. We chose the THP-1 cell line as a reference group because in our study we needed a control with stable characteristics. THP-1 cell line were the most convenient, accessible, and generally accepted model for studying the vascular system and CVD. In our work, we used macrophages as an internal control, which can be used to judge the adequacy of the methods used.

The aortic wall samples obtained during the operation of the aortic prosthetic were transferred by the staff of the First Cardiac Surgery Department of the Petrovsky National Research Center of Surgery. The separation of the aortic layers was performed mechanically under aseptic conditions. At first, we separated the tunica adventitia from the tunica media. Next, the sample with two layers (the tunica media and the tunica intima) was cut into two equal pieces. After that, to isolate SMCs from the tunica media, we put the first piece of the medial layer into the collagenase, and to isolate the SMCs from the tunica intima, we put the second piece of the inner layer into the collagenase. Primary cell cultures were isolated from the layers of the human thoracic aorta using collagenase type I (2 µg /ml) (STEMCELL, Cambridge, UK)^62,63^. Primary cells were seeded on 24-well plates in 0.6 ml DMEM/F12 (Gibco, Thermo Fisher Scientific, Waltham, Massachusetts, USA) with 10% fetal bovine serum (FBS) (Biowest, France), Penicillin–Streptomycin (STEMCELL, Cambridge, UK), and 2 mM L-glutamine (Thermo Fisher Scientific, Waltham, Massachusetts, USA) in a 5% CO_2_ humidified atmosphere at 37 °C. In our study we used cells at passages 2–7.

### Immunocytochemistry

For immunophenotyping, primary SMCs are labeled with antibodies directed against proteins: smooth muscle actin ACTA2 (1:100, Abcam, Cambridge, UK, ab220179), calponin CNN1 (1:100, Cloud-Clone Corp., Houston, Texas, USA, PAJ419Hu01), myosin heavy chain 11 MYH 11 (1:100, Cloud-Clone Corp Houston, Texas, USA, PAD420Hu01) according to the manufacturer’s protocol. We used secondary antibodies: Goat Anti-Mouse IgG H + L (PE) (1:100, Abcam, UK, № ab97024) and Goat Anti-Rabbit IgG H + L (FITC) (1:100, Abcam, UK, ab6717) to detection primary antibodies. We stained the nucleus with DAPI (BioFroxx, Germany, 28718-90-3). For the mounting of glasses, we used an Aqueous Mounting Medium (Abcam, Cambridge, UK, ab128982). We performed the fluorescence analysis on Leica DM4000 B LED (Leica Biosystems, Germany) and related software LAS-AF viewer Version 3.1.0 build 8587.

To study the phagocytic activity primary SMCs were seeded on 24-well plates (600 thousand cells in a well) in 0.6 ml DMEM/F12 with 10% FBS. THP-1 cells were grown in RPMI-1640 (Gibco, Thermo Fisher Scientific, Waltham, Massachusetts, USA) and supplemented with 10% FBS, Penicillin–Streptomycin, and 3.6 μL 2-Mercaptoethanol (Merck, Darmstadt, Germany) in a 5% CO_2_ humidified atmosphere at 37 °C. THP-1 monocytes can be differentiated to macrophages by incubation with 200 ng/mL PMA (Thermo Fisher Scientific, Waltham, Massachusetts, USA) for 2 days.

### Incubation with latex beads and LDL

Further, the studied cells were incubated with various agents:

1) incubation with latex beads FluoSpheres carboxylate, yellow-green 505/515, 0.5 µm (1:1000, Thermo Fisher Scientific, Waltham, Massachusetts, USA, F8803)^64^. We added latex beads to cells in DMEM/F12 without FBS and incubated for 3 h in a 5% CO_2_ humidified atmosphere at 37 °C. After incubation, study cells were washed three times with PBS solution to remove latex beads from the cells surface. The effectiveness of washing cells from latex particles was monitored by using a microscope Zeiss Axiovert 40 CFL (Zeiss, Germany). After washing, we used 0.25% trypsin solution (PanEco, Russia) for removing cells from the plate surface. Then the study cells were seeded on 3.5 cm coverglass bottom dishes with 50,000 cells in 150 µL of medium with 10% FBS per dish and incubated 24 h in a 5% CO_2_ humidified atmosphere at 37 °C. Then cells were fixed with a 4% PFA solution (Himedia, Kennett Square, PA, USA) and the nucleus were stained with DAPI. The obtained specimens were analyzed using a fluorescence microscope Leica DM4000 B LED (Leica Biosystems, Germany). We used three channels: DAPI, FITC, PMC and then we combined them using the appropriate software LAS-AF viewer Version 3.1.0 build 8587 for visualization. The analysis of the obtained images was carried out using the software «ImageJ». The proportion of cells containing latex beads in the cytoplasm was determined in the studied cultures. On each specimen, the number of absorbed microspheres in 10 cells in 5 fields (n = 50) of view was calculated with a 200-magnification microscope. The phagocytic activity of the cells was assessed by the number of beads per cell, then the number of absorbed latex beads per area of the studied cells was normalized.

2) incubation with atherogenic LDL^65^. LDL was isolated from the blood of patients with CVD. LDL was obtained by ultracentrifugation according to the previously described protocol^66^ LDL (100 µg/ml) was added to cells in a medium without FBS and incubated for 24 h. The control group of cells was incubated in a medium without FBS and LDL. After that, we used 0.25% trypsin solution for removing cells from the plate surface, and study cells were seeded on 3.5 cm coverglass bottom dishes with 50,000 cells in 150 µL of medium per dish and incubated 24 h in a 5% CO_2_ humidified atmosphere at 37 °C. Then cells were fixed with a 4% PFA solution. Accumulation of lipid droplets in cells was assessed using a fluorescent lipid dye BDP 630/650 (5мM) (Lumiprobe, Russia, 1233)^67^. All cell lines were stained with BDP 630/650 (Lumiprobe, Moscow, Russia) after fixation in PFA for 20 min at 37 °C in the dark, followed by six washes with PBS solution. The fluorescence intensity of the BDP 630/650 dye was measured in the studied cells in control groups and after incubation with LDL (n = 50 for each group) using the ImageJ program.

In our work, we studied 3 groups of study cells:

1. Group of cells after incubation with LDL;
2. Group of cells after incubation with latex beads;
3. Group of cells without agents – control group.

### RNA isolation and reverse transcription quantitative real-time polymerase chain reaction (RT-qPCR)

The isolation of total RNA from cultured cell lines was carried out using a kit for isolating total RNA based on the Lyra reagent (Biolabmix, Russia). To remove genomic DNA residues, the concentration of total nucleic acids was adjusted to 200 µg/ml, after that, a tenfold DNase buffer containing MgCl₂ (Life Technologies, USA) and 1 µl of DNase (Invitrogen, Thermo Fisher Scientific, USA) was added, and the mixture was incubated at 37 °C for 30 min. The concentration and purity of the obtained RNA were determined using an Implen NanoPhotometer N60 (Implen, Germany).

For reverse transcription, the M-MuLV–RH First Strand cDNA Synthesis Kit (Biolabmix, Russia) was used. In total, 6 µL (5 μg) of RNA was mixed with 3 µL of oligo (dT) and 3 µL deionized water, and incubated for 3 min at 70 °C. Further 4 μL of 5X × RT buffer mix (Biolabmix, Russia), 1 μL of 10 mM M-MuLV –RH revertase (100 u/µl) (Biolabmix, Russia) and 3 µL of deionized water were used for the reverse transcription reaction. Incubation continued for 60 min at 42 °C.

The expression of genes in the studied cell lines was determined by qPCR (Real-Time PCR) on a LightCycler 96 (ROCHE, Germany). For amplification, we used gene-specific primers: *ACTB* (actin beta), *GAPDH* (glyceraldehyde-3-phosphate dehydrogenase), *LDLR* (the low density lipoprotein receptor), *C3*(Complement component 3), *C4B* (Complement Component 4B) and the reaction mixture 5X qPCRmix-HS SYBR (Evrogen, Russia). The primers were ordered from Lumiprobe (Lumiprobe, Russia). Primer sequences: for *ACTB*—forward (5’-CACCATTGGCAATGAGCGGTTC-3’) and reverse (5’ AGGTCTTTGCGGATGTCCACGT-3’), for *GAPDH* forward (5’-ACTTTGGTATCGTGGAAGGACT-3’) and reverse (5’-GTAGAGGCAGGGATGATGTTCT-3’), for *LDLR* forward (5’-GGTCCAGTAGATGTTGCTGTGG-3’) and reverse (5’-GAATCTACTGGTCTGACCTGTCC-3), for *C3* forward (5’-GTGGAAATCCGAGCCGTTCTCT-3’) and reverse (5’-GATGGTTACGGTCTGCTGGTGA-3’), for *C4B* forward (5’-AGATGCGGTGTCCAAGGTTCTG-3’) and reverse (5’-GTTGCCAGGTATTTCCAAGGTCC-3’). The specificity of the primers was tested using the Primer-BLAST (NCBI) service.

qPCR reactions were performed in a total volume of 25 μL using 2 μL of cDNA (100 ng), 2 μL of forward and reverse primers (0.4 µM), 5 μL of qPCRmix-HS SYBR and 16 μL of deionized water.

qPCR was performed with pre-denaturation for 5 min at 95 °C, the next 40 cycles included denaturation for 15 s at 95 °C, annealing for 30 s at 62 °C and elongation for 30 s at 72 °C. For each sample, the qPCR reaction was performed at least three times.

The relative expression of target genes (*LDLR, C3* and *C4B)* in the studied cell lines was calculated using the formula: 2^−ΔΔ*Ct*^. *ACTB* and *GAPDH* were used as reference genes.

### ELISA

The conditioning cell culture media was removed from every well of plates for enzyme-linked immunosorbent assay (ELISA). Using ELISA, we measured the concentrations of IL-6, IL-8, TNF-α and IL-10, as they are the main markers of inflammation in aortic aneurysm^68–70^. The concentration of proinflammatory (IL-6, IL-8 and TNF-α) and anti-inflammatory (IL-10) cytokines in culture fluid samples was determined by DuoSet ELISA kits (R&D Systems, USA). ELISA was performed according to the manufacturer’s protocol. During the study, we analyzed three cell lines, each including three groups. For each cytokine, experiments were performed at least three times.

### Statistical analysis for BDP intensity, latex beads phagocytic activity, PCR and ELISA

Statistical data analysis was performed using the Python programming language and SciPy, sci-kit-learn libraries. Statistical significance was assessed using the Kruskal–Wallis test and the Conover multiple comparison test. T-test was used for pairwise comparison. For all results, p < 0.05 was considered significant. All values have been normalized by scaling to minimum and maximum values (min–max scaler).

### Liquid chromatography–mass spectrometry (LC–MS)

The conditioning cell culture media was removed from every well of plates for LC–MS. During the study, we analyzed three cell lines, each of which included three groups. Accordingly, there were nine measurements for each patient.

### Sample preparation

The reduction, alkylation, and digestion of the proteins were performed as described previously^71^ with minor modifications. Briefly, 10 µl of sodium deoxycholate (SDC) reduction and alkylation buffer pH 8.5 contained 100 mM TRIS, 1% (w/v) SDC, 10 mM TCEP, and 20 mM 2-chloroacetamide were added to a 10 µg protein sample. The sample was sonicated in an ultrasonic water bath for 1 min, heated at 85 °C for 10 min, cooled to room temperature, and the equal volume of trypsin solution in 100 mM TRIS pH 8.5 was added in a 1:50 (w/w) ratio. After overnight digestion at 37 °C, peptides were acidified by 50 µl of 2% trifluoroacetic acid (TFA) mixed with 50 µl of ethyl acetate and loaded on SDB-RPS StageTips contained two 14-gauge SDB-RPS plugs, and the StageTip was centrifuged at 300 g until all solution went through the StageTip (typically 4 min). After washing the StageTips with a 100 µl of 1% TFA/ethyl acetate 1:1 mixture (2 times) and 50 µl of 0.2% TFA, peptides were eluted in a clean tube by 60 µl 60% acetonitrile/5% ammonia mixture using centrifugation at 300 g. The collected material was vacuum-dried and stored at − 80 °C. Peptides were dissolved in 20 µl of 2% acetonitrile/0.1% TFA and sonicated for 1 min prior to analysis.

Samples were loaded to a home-made trap column 50 × 0.1 mm, packed with Reprosil-Pur 200 C18-AQ 5 μm (Dr. Maisch, Germany), in the loading buffer (2% ACN, 98% H_2_O, 0.1% TFA) at 4 μl/min flow and separated at RT in a home-packed^72^ fused-silica column 300 × 0.1 mm packed with Reprosil-Pur C18-AQ 1.9 μm (Dr. Maisch, Germany) into an emitter prepared with P2000 Laser Puller (Sutter, Atlanta, GA, USA). Reverse-phase chromatography was performed with an Ultimate 3000 Nano LC System (Thermo Fisher Scientific, Waltham, Massachusetts, USA), which was coupled to the Orbitrap Tribrid Lumos mass spectrometer (Thermo Fisher Scientific, Waltham, Massachusetts, USA) via a nanoelectrospray source (Thermo Fisher Scientific, Waltham, Massachusetts, USA). Water containing 0.1% (*v*/*v*) FA was used as mobile phase A and ACN containing 0.1% FA (*v*/*v*), 20% (*v*/*v*) H_2_O as mobile phase B. Peptides were eluted from the trap column with a linear gradient: 3% B for 5 min; 3–25% B for 28 min, 25–40% B for 25 min, 40–60% B for 4 min, 60% B during 3 min, 60–99% B for 0.1 min, 99% B during 10 min, 99–2% B for 0.1 min at a flow rate of 500 nl/min. After each gradient, the column was reequilibrated with A for 10 min. MS data was collected in DDA mode. MS1 parameters were as follows: 60 K resolution, 350–1600 scan range, max injection time—auto, AGC target—standard. Ions were isolated with a 1.2 m/z window, preferred peptide match, and isotope exclusion. Dynamic exclusion was set to 20 s. MS2 fragmentation was carried out in HCD mode at 15 K resolution with HCD collision energy 30%, max injection time—80 ms, AGC target—standard. Other settings: charge exclusion—unassigned, 1, > 6.

### LC–MS data analysis

Raw spectra were processed using MaxQuant 2.4.2.0 (MQ)^73^ and Perseus 2.0.3.1^74^. The data were searched against Human Uniprot SwissProt database, containing canonical proteins, version from 05.2023. MaxQuant search was performed with the default parameter set, including Trypsin/p protease specificity, max 2 missed cleavages, Met oxidation, protein N-term acetylation and N/Q deamidation as variable modifications, and Carbamidomethyl Cys as a fixed modification, max 5 modifications per peptide, 1% PSM and protein FDR. The following options were turned on: second peptide, maxLFQ, match between runs. All the runs were analyzed as independent experiments.

The protein group results were filtered for contaminants, reversed, and “identified only by site” proteins. In order to filter out the proteins that occurred in the cell culture medium upon lysis of dying cells we used an approach that was previously published^75^. Briefly, secretory proteins were filtered using UniProt keyword annotations as well as SecretomeP 2.0 predictions (10.1093/protein/gzh037):

- Proteins that were found to have “Signal” based on the UniProt keyword annotation were grouped as classical secreted proteins.
- The remaining proteins were annotated using GO Cellular Component (GOCC). Proteins that were found in the extracellular environment, such as in vesicles, according to the database were classified as non-classical secreted proteins. Of the remaining proteins, those that were found only in the intracellular environment (GOCC) were removed.
- The protein sequences for the remaining proteins were analyzed using SecretomeP. Proteins with SecretomeP NN-score > 0.5 were grouped as non-classical secreted proteins 2.0.

The resulting list of proteins was used for further statistical analysis in Perseus. Only the proteins with valid maxLFQ values in all 3 samples in at least one group were used. Missing values were imputed from normal distribution with 0.3 intensity distribution sigma width and 1.8 intensity distribution center downshift. Two-sample t-test with permutation-based FDR 5% was applied to search for significantly changing proteins, for multiple comparisons we used ANOVA with Tukey’s HSD test. Gene set enrichment analysis was performed with pydeseq2, Hallmark, Reactome, KEGG, GO pathway databases were used for analysis. Visualization of the results was performed with standard Python libraries: Seaborn, Matplotlib.

## Data Availability

The data that support the findings of this study are not openly available due to reasons of sensitivity and are available from the corresponding author upon reasonable request. Data are located in controlled access data storage at Petrovsky National Research Center of Surgery (https://med.ru/).
